# STORM without enzymatic oxygen scavenging for correlative atomic force and fluorescence superresolution microscopy

**DOI:** 10.1088/2050-6120/aad018

**Published:** 2018-07-09

**Authors:** Liisa M Hirvonen, Susan Cox

**Affiliations:** Randall Centre for Cell and Molecular Biophysics, King’s College London, New Hunt’s House, Guy’s Campus, London SE1 1UL, United Kingdom

**Keywords:** STORM, localisation microscopy, correlative microscopy, AFM+STORM, fluorescent probes, imaging buffers

## Abstract

Superresolution microscopy based on localisation is usually performed in a buffer containing enzymatic oxygen scavenger, which facilitates reversible photoswitching of the dye molecules. This makes correlative fluorescence localisation and atomic force microscopy (AFM) challenging, because enzymatic oxygen scavenging interferes with the AFM cantilevers. Here we report on the blinking kinetics of a new red cyanine dye, iFluor-647, which is similar to the Alexa-647 dye commonly used for superresolution microscopy, but with brightness and blinking properties which are superior to Alexa-647 in a buffer without enzymatic oxygen scavenger. We measure the blinking behaviour of iFluor-647 in buffers with and without enzymatic oxygen scavenger with different thiol concentrations. We then apply this dye for correlative localisation and atomic force microscopy in a buffer without enzymatic oxygen scavenger, which allows acquisition of AFM and superresolution images without buffer change.

## 1 Introduction

Fluorescence super-resolution microscopy techniques based on localisation, such as photoactivated localisation microscopy (PALM) [1] and stochastic optical reconstruction microscopy (STORM) [2] rely on the ability to switch the fluorophore between a bright and a dark state. In its most simple experimental form, direct STORM (dSTORM) [3, 4], the sample is illuminated with a high power laser while immersed in a reducing buffer which makes the molecules blink. The blinking efficiency depends strongly on the buffer; typically the removal of oxygen and addition of a thiol compound is required. Although this method does not allow much control over the switching kinetics, only one laser is required to achieve the transitions between the dark and the bright state as well as fluorescence excitation, making the experimental implementation relatively straightforward. The cyanine dye Alexa-647 has been widely reported as one of the best dyes for (d) STORM [3–5], with relatively high photon yield and low duty cycle in a buffer containing an enzymatic oxygen scavenging system and a thiol such as cysteamine (MEA) or *β*-mercaptoethanol (BME). The thiol is thought to form a covalent bond with the dye molecule, forming the dark state of cyanine dyes [6], and at moderate concentrations it can also act as an oxygen scavenger [7]. Despite its advantages [8, 9] and common use in STORM imaging buffers, oxygen scavenging changes the pH of the sample over time, degrading the sample and reducing the brightness of the dye.

Atomic force microscopy (AFM) [10] and fluorescence microscopy are a powerful combination in providing different types of information to complement each other [11, 12], and both are compatible with physiological buffers, allowing the observation of biological specimen in their natural environment. Fluorescence microscopy allows the tagging of intracellular molecules and cellular components with high specificity, and their observation inside cells in a minimally invasive manner using non-destructive wavelengths of light in the visible spectrum. AFM [10], on the other hand, uses a sharp tip to measure the topography of the sample with sub-nanometer axial resolution, or other physical properties of the surface, such as adhesion or stiffness. It is also possible to functionalise AFM tips to recognise specific molecules, and measure binding energies [11, 13], or use AFM for manipulation of the sample in nanometer scale [14]. Although the diffraction limit in light microscopy has restricted the resolution of fluorescence microscopy to two orders of magnitude more than AFM and made the correlation of these techniques difficult, recently developed superresolution microscopy techniques have brought the resolution of light microscopy down by an order of magnitude to few tens of nanometers, a similar scale to the typical lateral resolution of AFM when imaging soft biological samples [13, 15–17].

However, STORM imaging usually requires a switching buffer which contains enzymatic oxygen scavengers, typically glucose oxidase, catalase and glucose. Most attempts to combine AFM and STORM report adding the switching buffer after AFM imaging [18–21], as the buffer ingredients stick to the cantilevers used for AFM and make AFM image acquisition impossible. Although some fluorophores, including Alexa-647, have been reported to blink in a buffer without oxygen scavenger [5], the image quality usually suffers considerably. Moreover, in normal STORM buffer the pH changes over time, leading to detrimental changes in the dye molecule blinking properties and a typical maximum data acquisition time of ~2–3 h before the buffer has to be changed [22, 23], so there is a need for a STORM dye that can perform well in a buffer without an enzymatic oxygen scavenger.

An alternative approach for localisation microscopy that avoids buffers with enzymatic oxygen scavengers is the use of quantum dots (QDs) [19]. The tunable emission of QDs enables multi-colour localisation microscopy without the use of special buffers, however this approach requires a more sparsely labelled sample due to the long on-time of the QDs, and their bigger size of several nm in diameter can limit their use in labelling intracellular structures.

Here, we report on the switching kinetics of a fluorescent dye iFluor-647, which is especially suitable for imaging in thiol-only buffer, eliminating the need for enzymatic oxygen scavenger. iFluor-647 is a red cyanine dye similar to Alexa-647, with absorption peak at 654 nm and emission peak at 674 nm. Its quantum yield 0.25 in aqueous buffer is significantly increased when bound to proteins; the quantum yield of phalloidin-conjugated iFluor-647 is 0.65. We measure the brightness and photostability of Alexa-647 and iFluor-647 in thiol buffers with and without oxygen scavenger, test the image quality dependence on the buffer for STORM imaging, and demonstrate combined AFM+STORM imaging with iFluor in a single buffer without enzymatic oxygen scavenger.

## 2 Methods

### 2.1 Sample preparation

HeLa cells were cultured at 37 °C, 5% CO_2_ in DMEM supplemented with 10% FBS, penicillin/streptomycin, and L-glutamine. For blinking characterisation of the dyes, the cells were plated on 35 mm dishes with #1.5 glass coverslip bottom (WPI, FL) at seeding density of ~2 × 10^4^ cells per dish. The cells were left to adhere for 16–24 h, fixed for 20 min with 3.6% formaldehyde and permeabilised for 5 min in 0.1% Triton X-100. Alexa-647-phalloidin (A22287, Invitrogen, UK) and iFluor-647-phalloidin (23127, AAT Bioquest, CA) stock solutions were prepared according to manufacturer’s instructions. The stock solutions were diluted in 1% BSA in PBS, and the cells incubated for 30 min in the dye solution. For blinking characterisation, Alexa-647-phalloidin stock solution was used at 1 *μ*l/ml and iFluor-647-phalloidin stock solution at 0.04 *μ*l/ml concentration. For testing STORM image quality, Alexa-647-phalloidin was used at 25 *μ*l/ml and iFluor-647-phalloidin at 1 *μ*l/ml concentration.

For tubulin staining, HeLa cells were grown as above, but after permeabilisation the samples were blocked for 30 min in 3% BSA in PBS, incubated for 1 h with anti-*β*-tubulin mouse antibody (T8328, Sigma) diluted 1:200 in 3% BSA in PBS, washed thoroughly, and incubated for 1 h with either anti-mouse-Alexa-647 (A21235, Invitrogen) or anti-mouse-iFluor-647 (16783, AAT Bioquest) diluted 1:500 in 3% BSA in PBS.

For correlative AFM+STORM imaging, HeLa cells were plated on 35 mm dishes with #1.5 polymer coverslip bottom (ibidi, Germany) at seeding density of ~1.5 × 10^4^ cells per dish, and left to adhere for 16–24 h. To unroof the cells, the medium was replaced with H_2_O solution containing 10 *μ*g/ml phalloidin (sc-202763, Santa Cruz Biotechnology) and protease inhibitors (04693124001, Roche) for 40 s, the cells were then flushed 10× and fixed for 20 min with 3.6% formaldehyde. iFluor-647-phalloidin stock solution was diluted in 1% BSA in PBS at 2–4 *μ*l/ml concentration, and the cells incubated for 1 h in the dye solution.

### 2.2 Buffers

Stock solutions of MEA (1 M cysteamine (30070, Sigma-Aldrich) in H_2_O, pH adjusted to 8.0 with HCl solution) and GLOX (0.5 mg/ml glucose oxidase (G6766, Sigma-Aldrich), 40 *μ*g/ml catalase (C40, Sigma-Aldrich) in H_2_O) were stored at 4 °C and used within 1 week of preparation. The stock solutions were diluted in TN buffer (H_2_O with 50 mM Tris pH 8.0 and 10 mM NaCl; measured pH 7.7), supplemented with 10% w/v glucose if GLOX was added. GLOX stock was diluted 1:100, and MEA was used at final concentrations of 5–150 mM. For both MEA only and MEA+GLOX buffers the prepared buffer pH increased with the MEA concentration and varied between 7.8 for 10 mM MEA and 8.0 for 150 mM MEA. The buffers were mixed immediately before use and added to the sample dish 15–30 min before imaging. For buffers containing GLOX, the dish was covered with parafilm during imaging to reduce oxygen exchange.

### 2.3 Data acquisition

The samples were imaged with a standard inverted microscope (Zeiss Axio Observer.Z1). The microscope was equipped with a 647 nm laser (Cobolt 06-01 MLD 647) and an EMCCD (Andor iXon Ultra DU897_BV) for STORM data collection, and a JPK Nanowizard 3 for AFM imaging. For STORM, the sample was illuminated and imaged from the bottom through a 100X NA 1.4 oil immersion objective (Zeiss Plan-Apochromat) and a Cy5 filter cube (excitation 640/30 nm, dichroic mirror 660, emission 690/50 nm). For blinking characterisation, camera exposure time was 30 ms (28 Hz frame rate) and EM gain 300 with laser power at the sample ~14 kW cm^−2^. For STORM imaging, camera exposure time was 10 ms (60 Hz frame rate) and EM gain 400 (figure 2) or 600 (figure 3) with laser power at the sample ~5 kW cm^−2^. The camera pixel size at the sample plane was 145 nm, and the camera bit depth 16 bits. For characterisation 5,000 images were acquired of 64 × 64 pixel area of the sample, and three to five regions of interest were selected for each measurement. For imaging a total of 10,000 to 30,000 frames were acquired of areas between 64 × 64 and 128 × 128 pixels. The instrumentation for STORM data collection was controlled with *μ*Manager software [24].

For AFM imaging, a SiN cantilever with a Si tip with nominal spring constant of 0.292 N/m, tip radius <10 nm and gold coating on the reflex side (HYDRA-6V-200NG, Applied NanoStructures, CA) was mounted on the AFM head, and the head was placed on the top of the sample. Images were recorded on quantitative imaging (QI™) mode, which records a complete force-distance curve for each pixel without exerting lateral forces on the sample. For figure 3 the set point was 2 nN, and the scan time was ~16 min for 400 × 344 pixel image with 500 nm ramp size and 7 ms pixel time. The AFM images were processed by subtracting a 1st degree polynomial fit from each line.

According to UK research councils Common Principles on Data Policy, all data supporting this study is available on request from the authors.

### 2.4 STORM data processing

The raw STORM images were processed with ThunderSTORM [25] software using default processing parameters. For characterisation, the results were filtered to only select molecules with 50 nm < *σ* < 250 nm (where *σ* is the width of the fitted spot) and intensity >10 photons, and molecules appearing in consecutive frames were merged with merging radius of 150 nm and maximum 1 off-frame between detections. The mean molecule intensity was then calculated for each measurement. The number of molecules per frame was plotted as a rolling average of 20 frames against the accumulated exposure time since the start of the experiment, and the survival time calculated by fitting a double exponential function *y* = *α*_1_ · exp(−*x*/*τ*_1_) + *α*_2_ · exp(−*x*/*τ*_2_), where *α*_1_, *α*_2_ are the amplitudes and *τ*_1_, *τ*_2_ are the decay times, into the experimental data. The survival time (i.e. time before bleaching) was calculated as the average decay time $\tau =\left(\alpha_{1}\tau_{1}^{2}+\alpha_{2}\tau_{2}^{2}\right)/\left(\alpha_{1}\tau_{1}+\alpha_{2}\tau_{2}\right),$ and the amplitude-weighted survival time was calculated from 〈*τ*〉 = *α*_1_*τ*_1_ + *α*_2_*τ*_2_. For both intensity and survival time, the standard deviation (SD) was obtained from the variance of the 3–5 measurements of each sample. For testing dSTORM image quality (figure 2) some result images were corrected for drift using ThunderSTORM’s cross correlation function, but not filtered. For correlative AFM+STORM imaging (figure 3), the STORM images were post-processed to only select molecules with 50 nm < *σ* < 250 nm and intensity >10 photons, and molecules appearing in consecutive frames were merged with merging radius of 150 nm and maximum 1 off-frame between detections. Wide-field images are the standard deviation images of the raw image stacks.

## 3 Results

### 3.1 Single molecule brightness and blinking

**Figure 1:**
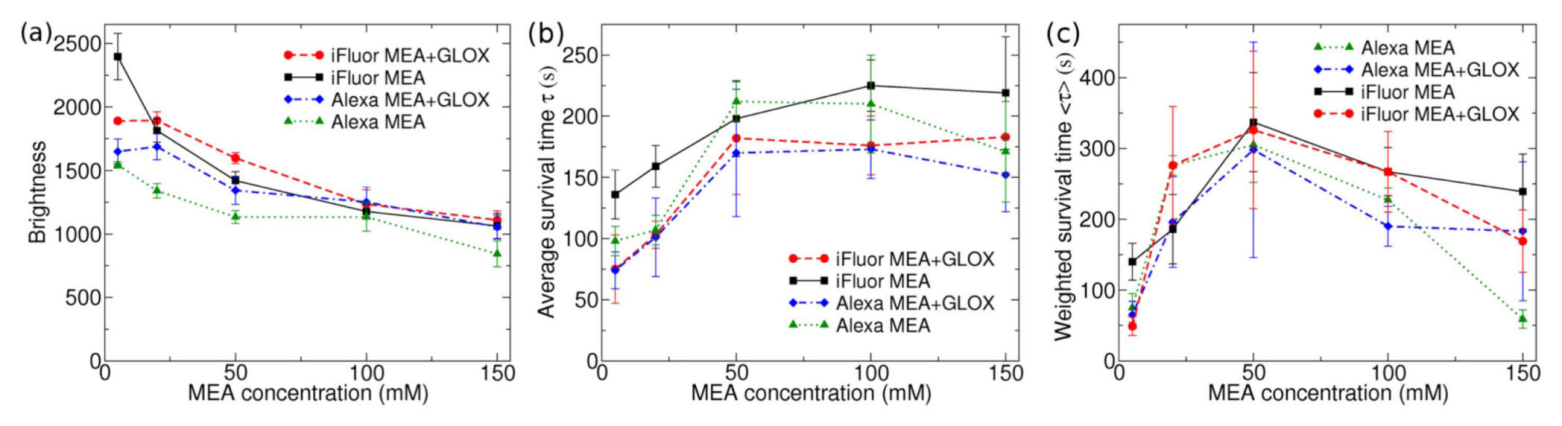
Results of single-molecule blinking characterisation for Alexa-647 and iFluor-647 dyes. (a) Mean molecule brightness, (b) average survival time, and (c) amplitude-weighted survival time as a function of MEA concentration. Error bars are standard deviations of 3–5 measurements.

First, the blinking properties of Alexa-647 and iFluor-647 were tested in different buffers. HeLa cells were grown on dishes with a glass coverslip bottom, and stained with a phalloidin conjugate of the dyes. The dye was used at a low concentration (1/25 manufacturer’s recommendation) to avoid overlapping molecules in the images. 5000 frames were collected for each data set, with 3–5 data sets for each sample. The frames of single molecule data were processed with ThunderSTORM [25], and the results were sigma-filtered to select only molecules on the focal plane based on the sharpness of the spot. The mean molecule brightness and mean survival time were then calculated for each sample (figure 1).

Consistent with previous reports [5], we found that Alexa-647 blinked poorly in PBS or in a buffer containing oxygen scavenger (GLOX) but no thiol, and reconstruction of a superresolution image was not possible. This was also observed for iFluor-647. With the addition of MEA as thiol, both Alexa-647 and iFluor-647 blink well in buffers with and without oxygen scavenger.

The brightness and survival time of both fluorophores were measured in MEA only and MEA+GLOX buffers as a function of MEA concentration. The brightness of the molecules was found to decrease with increasing MEA concentration, and both fluorophores were brighter in a buffer containing GLOX compared to an MEA only buffer: Alexa-647 was ~20 ± 7% and iFluor ~7 ± 4% brighter (figure 1(a)). For MEA concentrations of 20–50 mM, iFluor was 16 ± 3% brighter than Alexa in a buffer containing GLOX, and 30 ± 7% brighter in MEA only buffer (figure 1(a)). Importantly, the brightness of iFluor-647 in an MEA only buffer was comparable to the brightness of Alexa-647 in a buffer containing both MEA and GLOX. The brightness is also reflected in the localisation precision, with an overall decrease in localisation precision with increasing MEA concentration, and lower localisation precision for Alexa-647 in MEA only buffer (supplementary figure S1(a) available online at stacks.iop.org/MAF/6/045002/mmedia).

To estimate how long the individual dye molecules can be imaged before they are photobleached, a double-exponential function was fitted into the average number of molecules per frame (see supplementary figure S2 for examples of fitted data). The survival time (i.e. time before bleaching) was calculated as the average decay time $\tau =\left(\alpha_{1}\tau_{1}^{2}+\alpha_{2}\tau_{2}^{2}\right)/\left(\alpha_{1}\tau_{1}+\alpha_{2}\tau_{2}\right),$ see figure 1(b). There is a 50% increase in the survival time when the MEA concentration is increased from 20 mM to 50 mM, after which the survival time does not change significantly for increasing MEA concentration. No significant difference was found between iFluor-647 and Alexa-647.

Another important aspect for imaging is the proportion of fluorophores that blink. An amplitude-weighted survival time was calculated from 〈*τ*〉 = *α*_1_*τ*_1_ + *α*_2_*τ*_2_ (see figure 1(c)), which takes into account the number of molecules that are blinking. Although the data sets cannot be compared directly due to different labelling efficiencies and number of molecules in the field of view, when using similar samples (as is the case here) the number of molecules can give some indication of the blinking efficiency. For both types of fluorophores and buffers, there is an increase in the amplitude-weighted survival time up to 50 mM, and then a decrease.

### 3.2 STORM image quality

**Figure 2:**
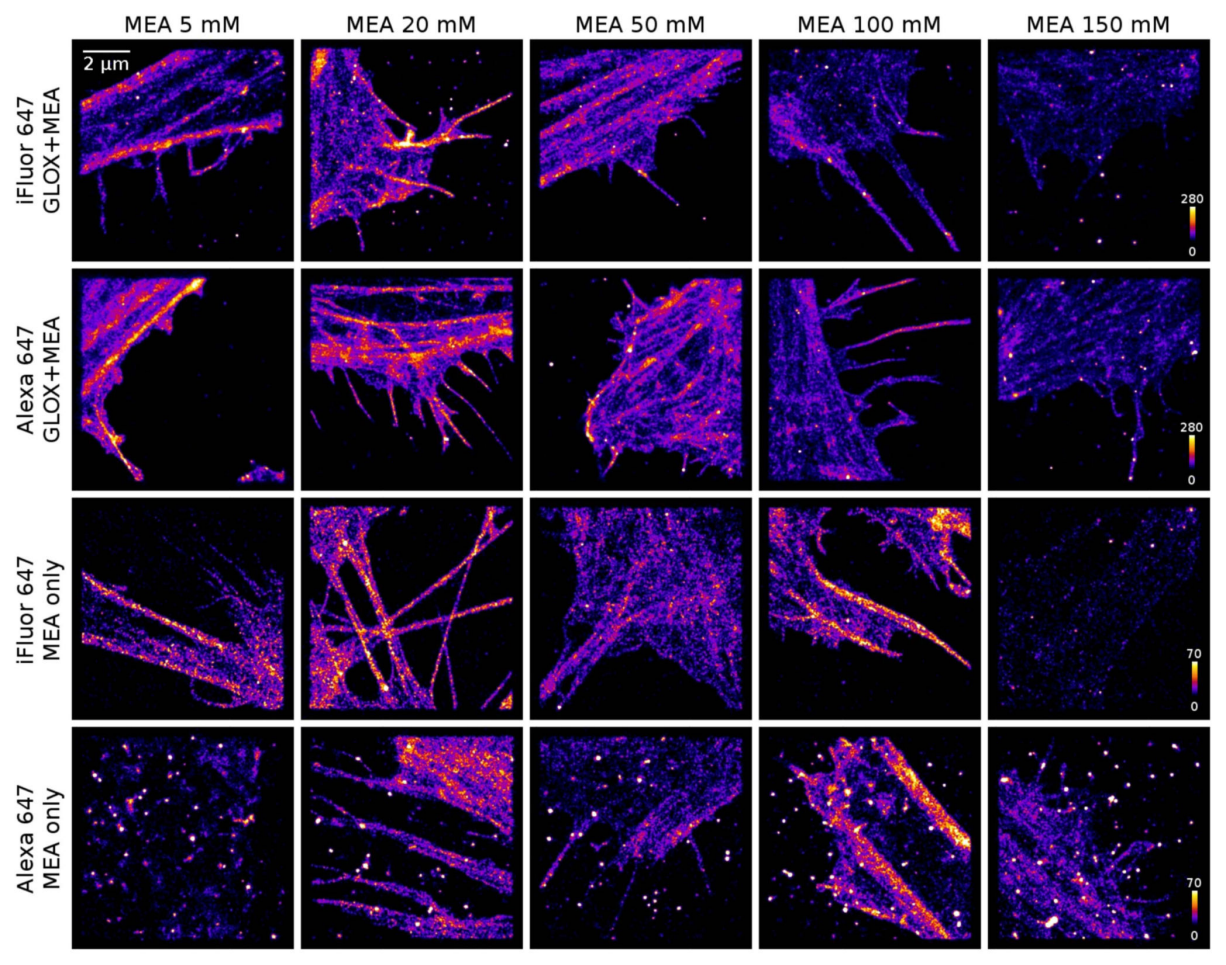
Representative images of HeLa cells stained with iFluor-647-phalloidin or Alexa-647-phalloidin in buffers with and without GLOX and with variable MEA concentration from 5 mM to 150 mM.

To test the STORM image quality, HeLa cells were labelled with Alexa-647-phalloidin and iFluor-647-phalloidin with manufacturer’s recommended dye concentration, and images were acquired in both MEA+GLOX and MEA only buffers with different MEA concentrations (see figure 2). For imaging, the duty cycle is also important—molecules that have high duty cycle (i.e. stay on for many frames with relatively low intensity in each frame) yield low quality images with bright spots in them, as the repeated localisation of the same dye molecule results in apparent clustering.

As expected, Alexa-647 performs well in a buffer containing both GLOX and MEA (figure 2, 2nd row), but in an MEA only buffer Alexa-647 yields overall poor image quality with bright spots in the localisation image (figure 2, bottom row). These spots are caused by background molecules with low number of emitted photons per frame and poor blinking properties; see supplementary figure S3. Although it is possible to reduce these clusters to some extent by merging reappearing molecules and filtering the results to only accept molecules with high enough brightness, this process also reduces the overall number of localised fluorophores in the image and therefore the image quality (see supplementary figure S4). iFluor, on the other hand, yields similar high quality images in MEA only buffer (figure 2, 3rd row) as Alexa-647 in MEA+GLOX buffer (figure 2, 2nd row). The average localisation precisions for both iFluor-647 in MEA only buffer and Alexa-647 in MEA+GLOX buffer are 14 ± 2 nm, whereas the lower photon yield of Alexa-647 in MEA only buffer is reflected in the 40% lower localisation precision of 20 ± 5 nm (supplementary figure S1(b)).

As expected from the amplitude-weighted survival time (figure 1(c)), the image quality degrades for MEA concentrations above 100 mM due to decreased photon yield and number of switching cycles. Interestingly, for both dyes the images acquired in the MEA+GLOX buffer have ~4× more localised fluorophores in areas with similar structure than the images acquired in the MEA only buffer; this difference seems to come from the proportion of molecules in the sample that blink. When using iFluor for combined AFM+STORM imaging, the dye concentration and incubation time were increased to compensate for this effect.

Besides phalloidin conjugates, iFluor-647 and Alexa-647 conjugated anti-mouse and anti-rabbit antibodies were also tested in MEA only buffer, and the performance was found to be similar to the phalloidin conjugates. Images of tubulin in HeLa cells stained with anti-mouse conjugates of the dyes are shown in supplementary figure S5.

### 3.3 Correlative imaging

For combined AFM+STORM imaging, unroofed HeLa cells were stained with iFluor-647-phalloidin, and before imaging the medium was changed to TN (Tris-NaCl) buffer with 50 mM MEA. When a suitable region of interest was found, the STORM and AFM images were recorded one after the other, as simultaneous acquisition is not practical due to the overlapping spectrum of the imaging and AFM laser wavelengths.

**Figure 3:**
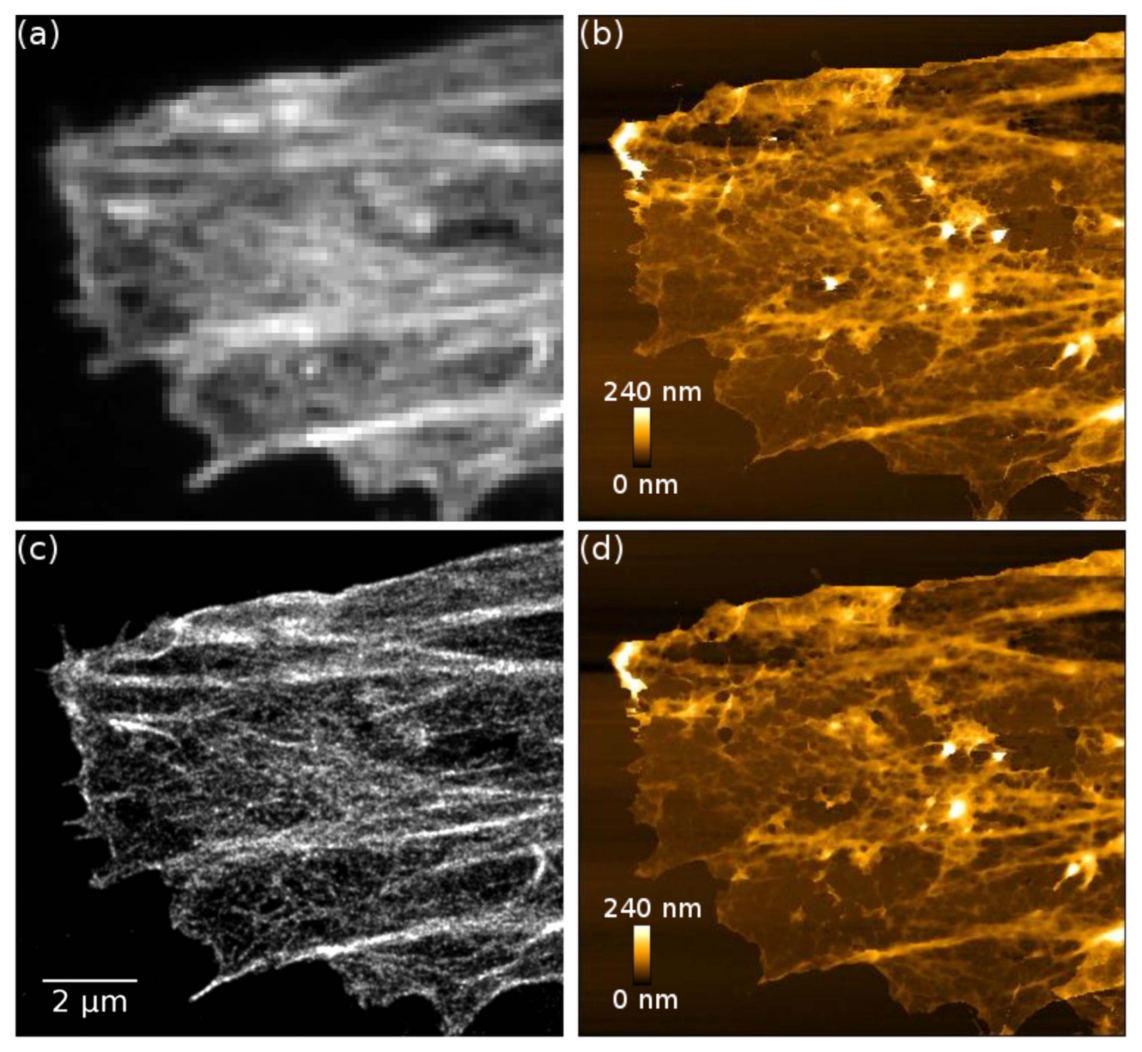
Correlative AFM and STORM images of an unroofed HeLa cell stained with iFluor-647-phalloidin. (a) Wide-field fluorescence image, (b) first AFM scan before STORM image acquisition, (c) STORM image, (d) second AFM scan after STORM image acquisition. The AFM image acquired after STORM acquisition (d) does not show observable damage compared to the AFM image acquired before STORM acquisition (b). AFM pixel size: 32 nm.

Figure 3 shows correlative AFM and STORM imaging of a HeLa cell. Here, an AFM image was acquired first (figure 3(b)), a STORM image was then acquired (figure 3(c)), and the area was then scanned again with AFM (figure 3(d)). No damage can be seen in the AFM image acquired after STORM acquisition.

The use of MEA only buffer also enables long term imaging. Supplementary figure S6(a)–(c) show an example of combined AFM+STORM imaging, where the STORM image was recorded first and the AFM image directly afterwards without the change of buffer. Supplementary figure S6(d)–(f) show an image of a different cell in the same sample after the sample has been in the microscope for >5 h without buffer change. Here the AFM image was recorded first, and the STORM image directly afterwards.

## 4 Discussion

Recent advances in both super-resolution microscopy and AFM have made combining these techniques a desirable tool for nanoscale biological research. A major drawback in combining AFM with localisation microscopy has been that the standard STORM buffer, containing enzymatic oxygen scavenger, is not compatible with AFM cantilevers. Buffer components, especially enzymes and glucose, interact with the cantilever and stick to the surface preventing AFM imaging (see supplementary figure S7) [20]. Because of this, the combination of STORM and AFM has usually required a buffer change between the imaging modalities [18–21], which is cumbersome and leads to longer time intervals and possible movement and damage to the sample between the images. Moreover, the pH change induced by the oxygen scavenger degrades the sample over time, leading to sample damage and limiting the imaging time to couple of hours.

In this work, we have tested a new red cyanine dye, iFluor-647, and compared the results with Alexa-647, the most commonly used dye for STORM imaging. We found that iFluor-647 molecular brightness is slightly higher than Alexa-647, particularly in an MEA only buffer, and the brightness of both fluorophores decreases with increasing MEA concentration (figure 1(a)), with the optimum MEA concentration for STORM imaging in the range of ~20–50 mM. When using these dyes for STORM imaging in MEA only buffer, some Alexa-647 molecules have a low duty cycle, leading to bright spots in the resulting images, whereas iFluor-647 yields good image quality in this buffer (figure 2). Alexa-647 also has lower brightness than iFluor-647 in MEA only buffer, which yields 40% lower localisation precision (supplementary figure S1(b)). Besides phalloidin conjugates, antimouse (supplementary figure S5) and anti-rabbit secondary antibody conjugated iFluor-647 and Alexa-647 dyes were also tested, with results similar to the phalloidin conjugates.

We have applied the iFluor-647-phalloidin conjugate for correlative AFM+STORM imaging of actin fibres in fixed HeLa cells in an MEA only buffer without enzymatic oxygen scavenger (figure 3, supplementary figure S6). The use of MEA only buffer enables correlative imaging without the change of buffer between the imaging modalities and without sample damage during long term imaging. Since there is no need for a buffer change, the AFM and STORM images can be acquired in whichever order is desired. Although some reports suggest that the AFM laser may bleach fluorescence in the red spectral region [18], we found that the 850 nm AFM laser in our system does not have significant effect on bleaching the 647 nm excitable fluorophores, and the STORM image quality is not compromised if the AFM image is acquired first. Some reports suggest that the STORM laser degrades the sample if the STORM image is acquired first so it could be beneficial to acquire the AFM image first [18, 20], however we found no evidence of sample damage after STORM imaging (figure 3); it is likely that the sample damage observed in MEA+GLOX buffer is diminished in MEA only buffer. Besides correlative AFM+STORM imaging, STORM imaging in MEA only buffer can be useful for any application where longer term (several hours) imaging of the sample is required, or sample damage caused by the STORM buffer is a concern.

## 5 Conclusion

We present an easy and straightforward method for correlative AFM+STORM imaging of fixed samples using iFluor-647 dye in a simple buffer containing the thiol MEA but no enzymatic oxygen scavenger. The brightness and blinking characteristics of iFluor-647 in an MEA only buffer are comparable to the popular STORM dye Alexa-647 in a buffer containing enzymatic oxygen scavenger, yielding good quality STORM images. Unlike buffers containing enzymatic oxygen scavengers, the use of MEA only buffer allows long term imaging over many hours and is compatible with AFM imaging such that no buffer change is required between the imaging modalities, simplifying the process and eliminating artefacts in correlative AFM +STORM imaging.

## Supplementary Material

Supplementary material for this article is available online

Supplementary Information
