# Supplementary material for: STORM without enzymatic oxygen scavenging for correlative atomic force and fluorescence superresolution microscopy
Source: Methods Appl Fluoresc. Author manuscript; Available in PMC 2019 May 29. (PMC6538533; doi:10.1088/2050-6120/aad018)
Supplement: Supplementary Information [file EMS83047-supplement-Supplementary_Information.pdf]

Supporting information: STORM without enzymatic oxygen scavenging for correlative atomic force and fluorescence superresolution microscopy

Liisa M. Hirvonen, Susan Cox

Localisation precision of characterisation data

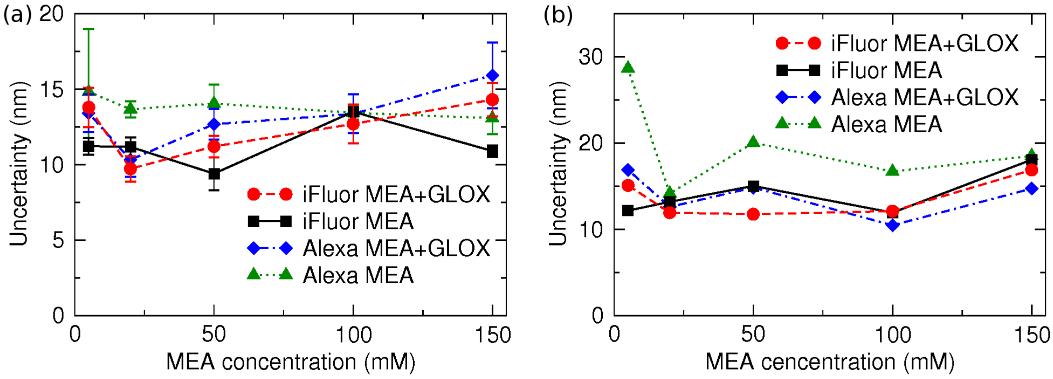

Figure S1: Localisation precision for data shown in (a) Figure 1 and (b) Figure 2. The plotted values are the median uncertainty for each data set obtained from fitting with ThunderSTORM. In (a) the error bars are the standard deviation from the measured 3-5 data sets. In (b) a maximum of 65,000 data points from each set were used for the calculation of the median.

Example fits for survival time calculation

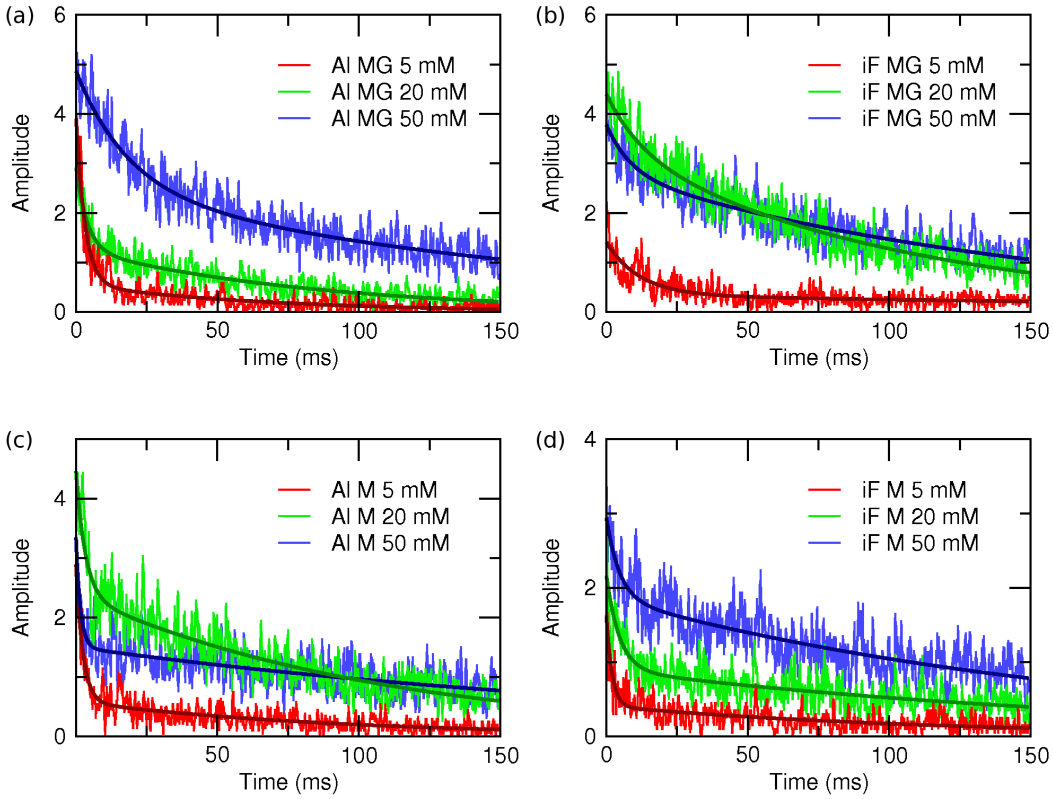

Figure S2: Examples of double-exponential fits to the number of molecules per frame (rolling average of 20 frames) as a function of time for survival time calculation. The decays were fitted with a double-exponential function $y = \alpha_1 \cdot \exp(-x/\tau_1) + \alpha_2 \cdot \exp(-x/\tau_2)$, where α_1, α_2 are the amplitudes and τ_1, τ_2 are the decay times. (a) Alexa-647 in MEA+GLOX buffer, (b) iFluor-647 in MEA+GLOX buffer, (c) Alexa-647 in MEA only buffer and (d) iFluor-647 in MEA only buffer.

Brightness of localised molecules

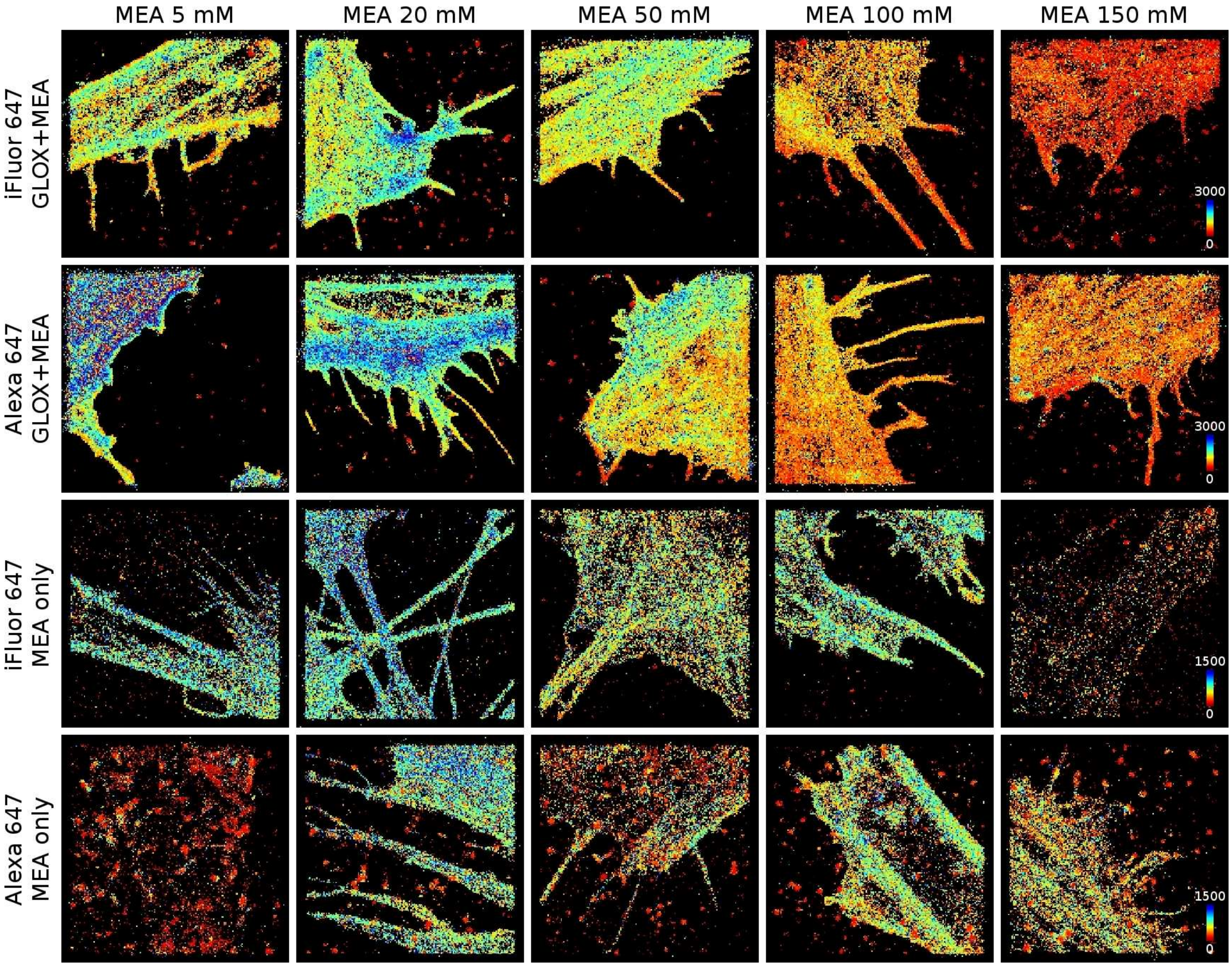

Figure S3: Molecule brightness in representative images of HeLa cells as shown in Figure 2. The colour represents the average number of photons per localised molecule in each pixel (red = low, blue = high). The molecule brightness decreases with increasing MEA concentration (from left to right), as expected from Figure 1. Furthermore, the bottom row for Alexa-647 in MEA only buffer shows many clusters of low-intensity molecules. These clusters correspond to the bright spots seen in Figure 2 for Alexa-647 in MEA only buffer, and are likely to be an artefact from repeated localisations of molecules with poor blinking properties.

Examples of post-processed images

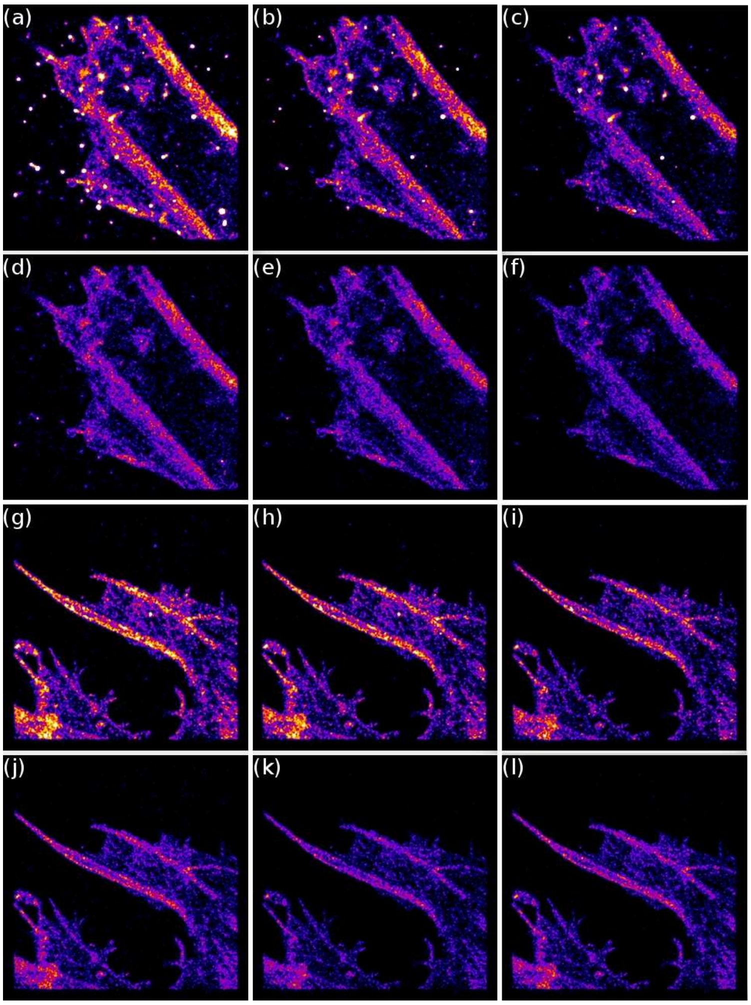

Figure S4: Examples of images post-processed with various methods to reduce the bright spots caused by molecules with low duty cycle. (a-f) Alexa-647 in 100 mM MEA only buffer, (g-l) iFluor-647 in 100 mM MEA only buffer. (a,g) No post-processing, and with post-processing filters (b,h) intensity $>$ 300 photons, (c,i) intensity $>$ 500 photons, (d,j) merged with 100 nm radius and max 30 off-frames, (e,k) intensity $>$ 300 photons and merged with 100 nm radius and max 30 off-frames, (f,l) intensity $>$ 500 photons and merged with 100 nm radius and max 30 off-frames. Filtering reduces the bright spots but also the brightness of the whole image.

Images of tubulin labelled with antibody-conjugated dyes

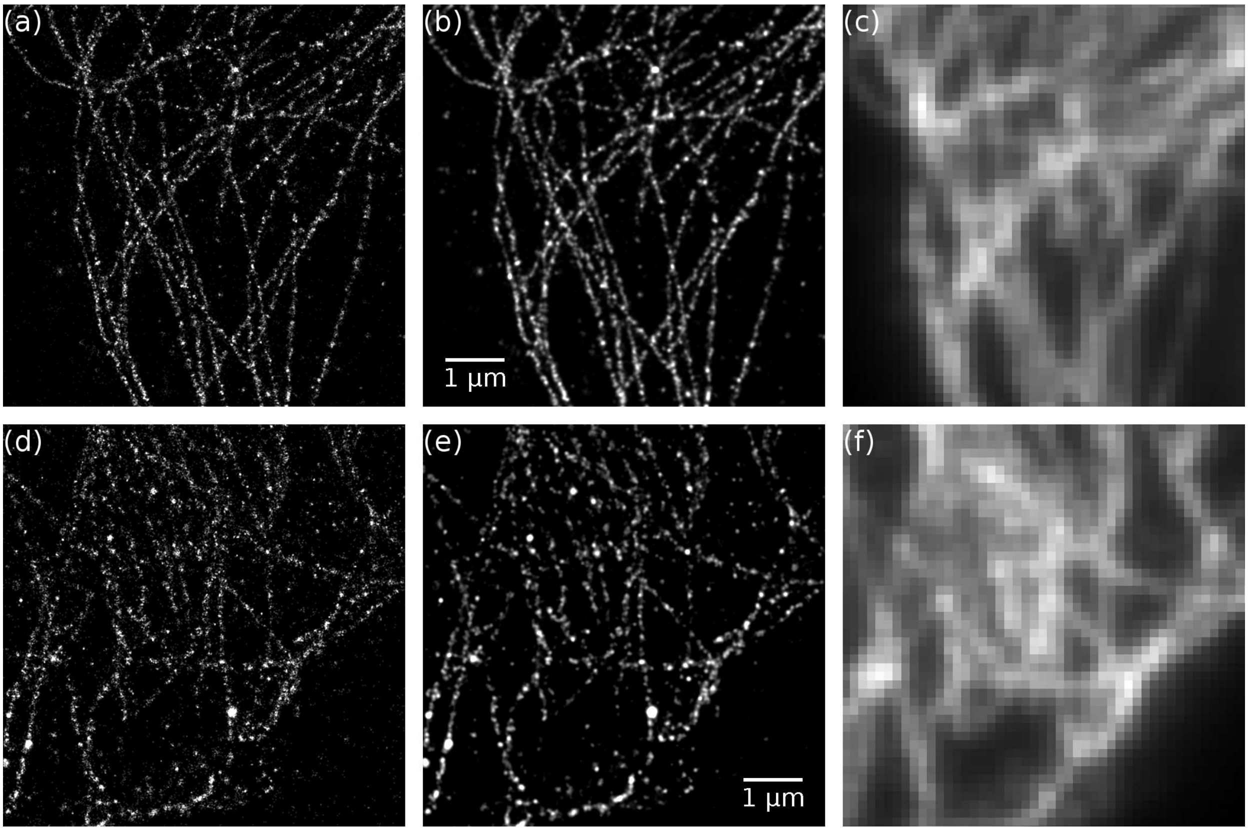

Figure S5: Example images of secondary antibody labelled tubulin in HeLa cells, stained with (a-c) iFluor-647 and (d-f) Alexa-647 and imaged in 50 mM MEA buffer (no GLOX). (a,d) STORM images, (b,e) STORM images with Gaussian blur, (c,f) wide-field images. In the iFluor images the tubulin fibres can be seen clearly, whereas the poor blinking characteristics of Alexa-647 lead to bright spots and broken fibres.

Correlative AFM+STORM images

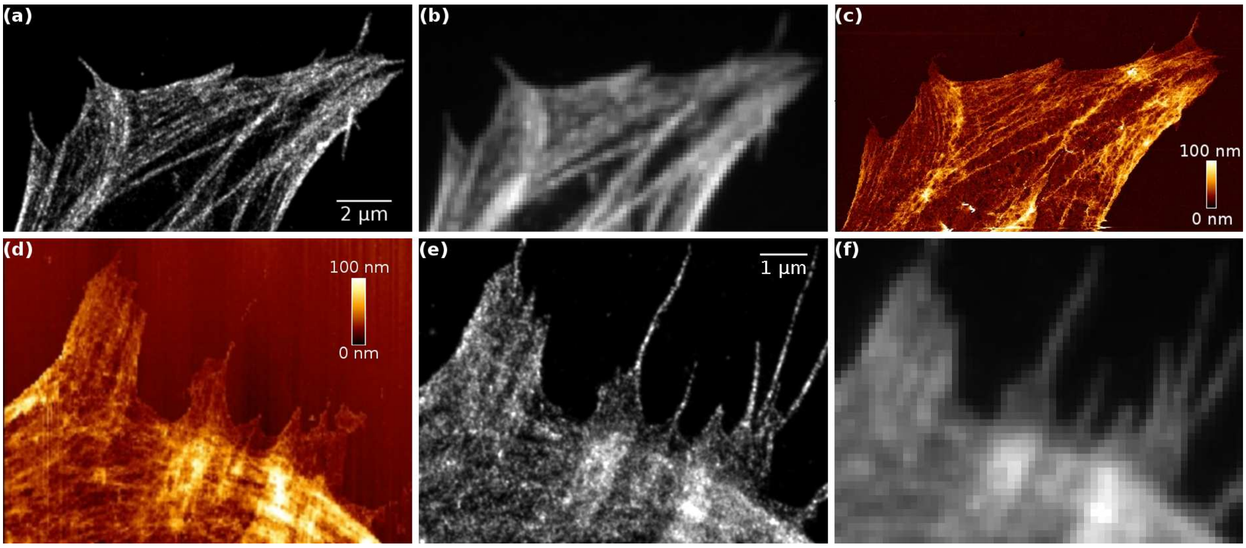

Figure S6: Correlative STORM and AFM images of of unroofed HeLa cells stained with iFluor-647-phalloidin. (a) STORM, (b) wide-field, and (c) AFM images, where the STORM image was acquired first in 50 mM MEA buffer, and the AFM image directly afterwards without buffer change. (d) AFM, (e) STORM and (f) wide-field images of a different area of the same sample after >5 hours in the microscope without buffer change; here the AFM image was acquired first, and the STORM image directly afterwards. AFM pixel size (c) 30 nm, (d) 40 nm. The set point was 4 nN for both images, and the scan time was ∼19 minutes for 512×284 pixel image with 800 nm ramp size and 8 ms pixel time (c) or ∼6 minutes for 256×256 pixel image with 500 nm ramp size and 5 ms pixel time (d).

Cantilever photos

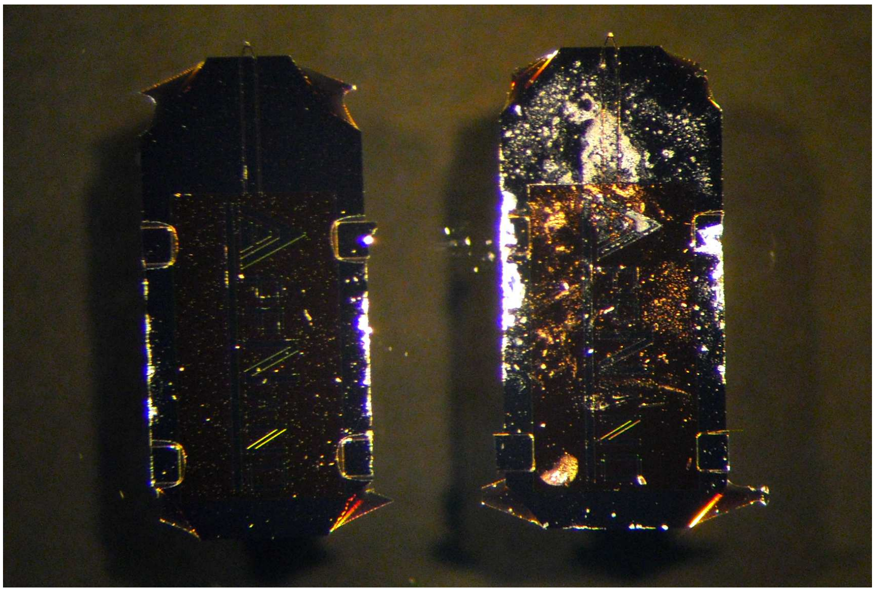

Figure S7: Photos of AFM cantilevers, before (left) and after (right) exposure to STORM buffer containing an oxygen scavenger. The cantilever looks "dirty" after being exposed to the switching buffer, a possible explanation is that the glucose in the buffer sticks to the cantilever and forms crystals.
